# Hysterectomy for Cancer of the Uterus

**Published:** 1900-11-24

**Authors:** 


### HYSTERECTOMY FOR CANCER OF THE UTERUS.

At the last meeting of the Royal Medical and Chirurgical Society Dr. Lewers read a paper on the after-results in forty consecutive cases of vaginal hysterectomy performed for cancer of the uterus. In twelve cases there had been no recurrence of the disease; in eighteen there had been recurrence; four cases had died; in two cases the nature of the disease could not be proved by examination of the specimens; in three no definite information was obtained as to recurrence; and in one the disease proved on examination to be primary tuberculous ulceration of the cervix. Eleven of the cases (or 27·5 per cent.) were remaining well from two to more than seven years after the operations. A careful selection of cases had been made and the operations had only been done when the disease appeared to be limited to the uterus itself. On the other hand, for some time past Dr. Lewers had performed supravaginal amputation for very early cases and for cancer of the vaginal portion in which there was only a limited and superficial growth. He had performed thirty three amputations of this kind, but these were not included in his paper. One of the conclusions arrived at by the author of the paper was that the great desideratum was early diagnosis. The discussion served to show how largely this operation is being resorted to by different operators in the treatment of cancer of the uterus. Thus Dr. Briggs, of Liverpool, said that he had had 83 cases with four deaths, Dr. Amand Routh had had 40 cases with 3 deaths, Mr. Bowreman Jessett 150 cases, and Mr. Walter Tate 70 cases; and if, as is probably the case, other gynæcologists are going on at the same rate, a very few years ought to throw much valuable light upon the prognosis in these miserable cases. We may add that exactly in proportion as cure, or even delay in recurrence, is shown to attend early operation, so will the responsibility which lies upon those whose duty it is to discover the disease at the earliest possible moment be increased.

Speaking in regard to this point Mr. Bowreman Jessett said that the point of prime importance was early diagnosis. If practitioners could but be impressed with the idea how important it was to see these cases early, he thought that the results would be better. As an early symptom, one even earlier sometimes than intermenstrual hæmorrhage, he insisted on the significance of hæmorrhage during coitus. Dr. Champneys spoke of the value of examination per rectum as a means of diagnosis, for in this direction one could reach higher, and palpate the parts around with greater facility, than in other ways. He thought that operation was not justifiable if the disease extended beyond the uterus, but it was often difficult to be certain about this. This paper, and the discussion which it elicited, will do much to fix the attention of the profession on the fact that cancer of the uterus taken early and removed thoroughly is by no means the hopeless affair that it used to be considered only a few years ago.

